# Supplementary material for: Three-Dimensional Co-Culture System of Human Osteoblasts and Osteoclast Precursors from Osteoporotic Patients as an Innovative Model to Study the Role of Nutrients: Focus on Vitamin K2
Source: Nutrients. 2021 Aug 17;13(8):2823. doi: 10.3390/nu13082823 (PMC8399348; doi:10.3390/nu13082823)
Supplement: Supplementary file 1 [file nutrients-13-02823-s001.zip › nutrients-1313905-supplementary.pdf]

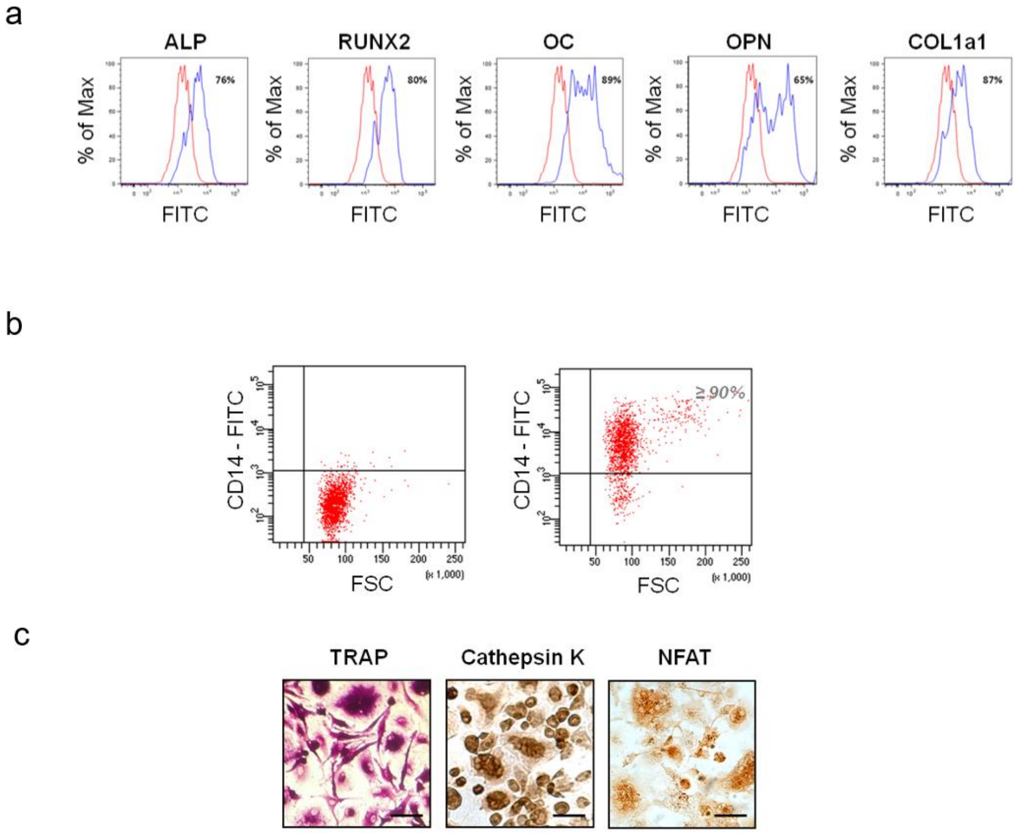

Supplementary Figure S1. Evaluation of isolated hOBs and hOCs quality after thawing. (**a**) Representative histogram of ALP, Runx2, OC, OPN and COL1a1 cytometric expression. (**b**) Representative dot plots of CD14+ hOCs. (**c**) Representative images of TRAP, Cathepsin K and NFAT immunohistochemistry analysis. FSC (Forward Scatter); FITC (Fluorescein isothiocyanate); ALP (Alkaline Phosphatase); RUNX2 (Runt-related transcription factor 2); OC (Osteocalcin); OPN (Osteopontin); COL1a1 (Collagen type 1 alpha); TRAP (Acid Phosphatase Leukocyte); NFAT (Nuclear factor of activated T-cells).
